# Toll-like Receptors in Viral Encephalitis

**DOI:** 10.3390/v13102065

**Published:** 2021-10-14

**Authors:** Olivia Luise Gern, Felix Mulenge, Andreas Pavlou, Luca Ghita, Imke Steffen, Martin Stangel, Ulrich Kalinke

**Affiliations:** 1 Institute for Experimental Infection Research, TWINCORE, Centre for Experimental and Clinical Infection Research, a Joint Venture between the Helmholtz Centre for Infection Research and the Hannover Medical School, 30625 Hannover, Germany; felix.mulenge@twincore.de (F.M.); andreas.pavlou@twincore.de (A.P.); lghita@stanford.edu (L.G.); ulrich.kalinke@twincore.de (U.K.); 2 Department of Pathology, University of Veterinary Medicine Hannover, Foundation, 30559 Hannover, Germany; 3 Clinical Neuroimmunology and Neurochemistry, Department of Neurology, Hannover Medical School, 30625 Hannover, Germany; 4 Center for Systems Neuroscience, University of Veterinary Medicine Hannover, 30559 Hannover, Germany; 5 Division of Infectious Diseases and Geographic Medicine, Department of Microbiology and Immunology, Stanford University School of Medicine, Stanford, CA 94305, USA; 6 Department of Biochemistry and Research Center for Emerging Infections and Zoonoses (RIZ), University of Veterinary Medicine Hannover, Foundation, 30559 Hannover, Germany; Imke.Steffen@tiho-hannover.de; 7 Translational Medicine, Novartis Institute for Biomedical Research (NIBR), 4056 Basel, Switzerland; martin.stangel@novartis.com; 8 Cluster of Excellence—Resolving Infection Susceptibility (RESIST, EXC 2155), Hannover Medical School, Carl-Neuberg-Straße 1, 30625 Hannover, Germany

**Keywords:** viral encephalitis, Toll-like receptors, CNS, viruses, neurons, astrocytes, microglia

## Abstract

Viral encephalitis is a rare but serious syndrome. In addition to DNA-encoded herpes viruses, such as herpes simplex virus and varicella zoster virus, RNA-encoded viruses from the families of Flaviviridae, Rhabdoviridae and Paramyxoviridae are important neurotropic viruses. Whereas in the periphery, the role of Toll-like receptors (TLR) during immune stimulation is well understood, TLR functions within the CNS are less clear. On one hand, TLRs can affect the physiology of neurons during neuronal progenitor cell differentiation and neurite outgrowth, whereas under conditions of infection, the complex interplay between TLR stimulated neurons, astrocytes and microglia is just on the verge of being understood. In this review, we summarize the current knowledge about which TLRs are expressed by cell subsets of the CNS. Furthermore, we specifically highlight functional implications of TLR stimulation in neurons, astrocytes and microglia. After briefly illuminating some examples of viral evasion strategies from TLR signaling, we report on the current knowledge of primary immunodeficiencies in TLR signaling and their consequences for viral encephalitis. Finally, we provide an outlook with examples of TLR agonist mediated intervention strategies and potentiation of vaccine responses against neurotropic virus infections.

## 1. Viral Encephalitis

Viral encephalitis is the pathological inflammation of the brain parenchyma, which is triggered by infection with a wide range of neurotropic RNA and DNA viruses. The syndrome is a rare condition, yet it may have serious clinical consequences. Despite medicinal progress that has been made during recent years, approximately 60% of presumed cases of viral encephalitis remain of unknown etiology [1]. The clinical manifestations of viral encephalitis are extremely variable and are influenced by the extent of inflammation, the range of viral dissemination and the specific brain regions that are affected. Generally, viral encephalitis is marked by acute fever, headache, altered level of consciousness, seizures and neurologic deficits. Often, survivors suffer from severe neurological and neuropsychological sequelae, while there exist only limited treatment options and preventive vaccines are only available for few neurotropic pathogens [2]. Among DNA viruses, the members of the Herpesviridae family herpes simplex virus and Varicella-zoster virus are the most relevant pathogens that account for the majority of sporadic encephalitis cases in humans worldwide. In developed countries, herpes simplex virus type 1 (HSV-1) is the leading cause of sporadic viral encephalitis in adults [3]. The incidence of herpes simplex encephalitis (HSE) is approximately 2.2 cases per one million people, with a mortality rate of more than 70% before acyclovir was introduced for treatment [4]. Even though all age groups can be affected, the disease incidence is more prominent, and the disease course more severe, in children and the elderly. HSV-1 gains access into the host through mucous membranes or damaged skin and establishes latency in the trigeminal ganglia [5]. Only upon reactivation of latent HSV-1 the virus can access the CNS. The CNS entry mechanism involves retrograde axonal transport via sensory neurons of the orofacial mucosa, whereas the mechanism of HSV-1 reactivation from latency is still largely unclear [6,7,8]. 

Varicella-zoster virus (VZV) is the second most common cause of sporadic viral encephalitis, accounting for 5% of all viral encephalitis cases in humans [9]. In the United States, VZV affects approximately 30% of people during their lifetime [10]. VZV encephalitis occurs with high incidence primarily in the elderly and in immunocompromised patients [11]. Primary infection is caused by exposure to infectious fomites from skin lesions and inhalation of infectious droplets. Initially, VZV undergoes replication in regional lymph nodes [12]. Subsequently, the virus disseminates in a retrograde manner via sensory neurons of the dorsal root ganglia and establishes lifelong latency. Advanced age and immunosuppression favor VZV reactivation in the dorsal nerve ganglia causing viral spread in a centripetal pattern (towards the brain), a centrifugal pattern (towards the skin) or both directions in an anterograde manner [13,14].

Recent epidemiologic data suggests that approximately 83% of the global population is seropositive for the herpesvirus human cytomegalovirus (HCMV), which establishes lifelong latent infection [15]. Immunocompetent individuals control the latent infection, but upon immunosuppression, aging, or during pregnancy, HCMV can be reactivated and disseminate to large parts of the body, including the CNS, then conferring high morbidity and mortality [16,17].

Highly pathogenic RNA viruses including members of the families Flaviviridae, Rhabdoviridae and Paramyxoviridae can cause encephalitis. Rabies virus (RABV) of the family of Rhabdoviridae induces lethal disease in 100% of unvaccinated subjects, manifesting symptoms as classic furious rabies and paralytic rabies. Annually, 60,000 human deaths are reported [18]. The virus infects peripheral nerves at the site of entry and travels in a retrograde manner by axonal transport to the neurons of the dorsal root ganglia. From there, the virus ascends axonally to the brain, where it leads to behavioral changes, seizures, and coma [19].

Within the Flaviviridae family, viruses that cause CNS infections include Japanese encephalitis virus (JEV), West Nile virus (WNV), tick-borne encephalitis virus (TBEV), Zika Virus (ZIKV) and dengue virus (DENV) [20]. Their prevalence and incidents vary geographically. Nevertheless, up to 400 million people are infected annually with DENV [21]. Development of flaviviral encephalitis is presumed to occur mainly through hematogenous spread of the virus. After virus entry at the site of a mosquito or tick bite, the virus replicates in local tissues, which results in primary viremia and subsequent infection of extraneural tissues that may eventually lead to further CNS infection [22]. Extensive investigations about how flaviviruses overcome the blood-brain barrier (BBB) during natural infection and gain access to the CNS revealed a plethora of mechanisms, such as active replication within the endothelial lining of the BBB as well as passive transfer across the BBB and “Trojan horse” migration [23,24,25], whereas several aspects still remain unknown.

In the 1990s, two extremely virulent members of the Paramyxoviridae family affecting the CNS emerged. The RNA-encoded Hendra- and Nipah viruses (HeV and NiV, respectively) are zoonotic pathogens that are found in bats and have primarily spilled over to horses or swine, respectively, but can also be transmitted to humans, where they lead to unforeseeable disease outcomes [26]. HeV spreads throughout various peripheral organs to the meninges and the blood vessels to eventually reach the brain, where it causes characteristic lesions [27]. Even though HeV infection in humans is extremely rare, with only seven reported cases to date [28], it harbours enourmous zoonotic potential and future outbreaks are likely to happen. NiV cases are more frequent with approximately 700 human cases in Southeast Asia reported until 2018 [29]. Both viruses are leading to serious illness in humans with a case-fatality rate of 60% for HeV infection [30], and 53% for NiV infection while NiV is highly neurotropic and affects several regions of the brain [29].

## 2. Toll-like Receptors and Toll-like Receptor Signaling

Viruses and microorganisms such as bacteria, parasites and fungi have evolved and diversified; however, a number of conserved fundamental traits are shared among different classes of infectious agents. These recurring pathogen-associated molecular patterns (PAMPs) are sensed through germline-encoded pattern recognition receptors (PRRs). The first PRRs to be discovered were Toll-like receptors (TLRs) in the drosophila system that were initially linked with developmental processes and later connected to immunity against pathogens [31,32]. In humans, the TLR family comprises TLR1–10, while in mice homologues of TLR1–9 exist and, additionally, TLR11–13 are present [33,34,35,36,37,38,39,40,41,42,43,44]. TLR1, 2, 4, 5, 6 and 10 are localized primarily on the cell surface and mostly recognize extracellular bacterial components, whereas TLR2 and TLR4 have been shown to contribute to the detection of viral surface proteins as well [45,46,47,48]. In contrast, TLR3, TLR7–9 and TLR11–13 are localized in intracellular compartments such as endosomes, whereas TLR4 is constitutively expressed on the cell surface and, with support of CD14, can be endocytosed upon ligand binding [49,50]. Especially, foreign nucleic acid species of viral or bacterial origin can be sensed by these intracellular TLRs, which are of tremendous importance for virus detection and initiation of protective responses [51]. Besides TLRs, other sensing platforms exist, such as RIG-I like receptors (RLRs) [52,53,54,55,56,57], cyclic GMP AMP synthase (cGAS) [58,59], NOD-like receptors (NLRs), which constitute the inflammasome [60,61], AIM-2 like receptors (ALRs) [62], and C-type lectin receptors (CLRs) [63]. In this review, we will focus on the role of TLRs in viral encephalitis.

Upon binding of the corresponding ligands, TLR homo- or heterodimers are formed [64]. Consequently, a signaling cascade is triggered, involving an intracellular web of adaptor molecules and transcription factors leading to the expression of pro-inflammatory factors. Most TLRs, except for TLR3 and TLR10, recruit the adaptor myeloid differentiation primary response 88 (MyD88). TLR3, located in the endosomes, as well as endocytosed TLR4, recruit the adaptor TIR-domain-containing adapter-inducing interferon-β (TRIF) [65,66]. For TLR10, the adaptor molecule remains unknown and current data point towards a rather anti-inflammatory effect, suggesting the involvement of a different adaptor than MyD88 or TRIF [67]. Upon activation, MyD88 and TRIF induce complexes of IL-1R-associated kinases (IRAKs), TNF receptor-associated factors (TRAFs) and TANK-binding kinase 1 (TBK1). Finally, transcription factors such as nuclear factor-κB (NF-κB), cyclic AMP-responsive element-binding protein (CREB), activator protein 1 (AP1) and interferon-regulatory factors (IRFs) are engaged, leading to transcriptional activity of gene loci encoding type I interferons (IFN-I) and other pro-inflammatory cytokines [65].

Type I IFNs, such as IFN-α and β, are expressed following TLR signaling and act in an auto or paracrine manner through the IFN-I receptor (IFNAR), that consists of the heterodimer of IFNAR-1 and IFNAR-2. This leads to the initiation of an intracellular signaling cascade via Janus kinase 1 (JAK1) and tyrosine kinase 2 (TYK2). Upon JAK1 and TYK2 phosphorylation and activation, the signal transducers and activators of transcription 1 and 2 (STAT1 and STAT2) are triggered and associate with IRF9. This complex of STAT1, STAT2 and IRF9 binds to IFN-stimulated response elements (ISRE) in the genome and launches activation of the transcription of hundreds of IFN stimulated genes (ISGs), which results in the establishment of an antiviral state [68].

The significance of TLR signaling during viral encephalitis has been highlighted by primary human immunodeficiencies in the TLR3 pathway, as well as by in vivo studies with several genetically modified mouse lines that were deficient in selected TLRs or adaptor molecules [69,70,71,72,73]. In the following, the knowledge gained through mouse models with global TLR deficiencies is recapitulated. 

Studies on the role of TLR3 during West Nile virus infection revealed controversial results as to its protective role and on its impact on the permeability of the BBB. Indeed, the kind of effect could be associated with the number of in vitro passages of WNV, as well as the route of infection and the dosage [71,73]. Furthermore, TLR3 was shown to regulate BBB leakage and to contribute to host protection during JEV infection [74]. Interestingly, TLR3^‒/‒^ mice showed impaired survival upon TMEV intracerebral infection, as shown by spatiotemporal triggering of TLR3 signaling that is responsible for distinct outcomes of T cell activation and immune cell CNS infiltration [75]. TLR3^‒/‒^ mice showed enhanced sensitivity to intracerebral HSV-1 infection, which was reminiscent of the phenotype detected in HSE patients with primary defects in the TLR3 pathway [70]. Potentiation of TLR3 signaling by administration of an agonistic anti-TLR3 monoclonal antibody (mAb) rescued mice from lethal HSE [70]. Similarly, intranasal HSV-1 instillation of TRIF^‒/‒^ mice revealed the importance of TLR3 signaling for protection against HSE. TRIF^‒/‒^ mice displayed marked viral infection of the olfactory bulb and delayed induction of IFN-I responses when compared with infected WT mice [72]. The enhanced susceptibility of TLR3^‒/‒^ and TRIF^‒/‒^ mice could be further explained by the direct interaction of TRIF with STING that promotes STING dimerization and thus induces subsequent signaling, which is essential for protection against lethal HSE [76,77,78]. However, more in vivo functional data are needed to fully understand the interaction between TRIF and STING. Enhanced neuroinvasiveness and increased infection susceptibility were also detected in an intravaginal HSV-2 infection model of TLR3^‒/‒^ mice [79], further highlighting the importance of TLR3 and its downstream signaling in host protection against HSE.

The adaptor molecule MyD88 is highly relevant for the control of neurotropic viruses and the restriction of their spread within the CNS, as shown in experimental setups with WNV and vesicular stomatitis virus (VSV), the latter of which belongs to the family of Rhabdoviridae, related to RABV. MyD88^‒/‒^ mice show enhanced sensitivity to peripherally administered WNV and to intranasally instilled VSV, with a pronounced inability to restrict viral replication within the CNS parenchyma [80,81,82]. Interestingly, MyD88 mediated protection from VSV is due, to a large extent, to MyD88 signaling in neurons, since cell type-selective reconstitution of MyD88 only in the neuronal compartment sufficed to revert the susceptibility of complete MyD88 deficiency upon intranasal VSV instillation [80]. The MyD88 mediated protection from lethal VSV infection was mediated by neuronal chemokine production [80,81]. Along that line, double MyD88 and TRIF deficient mice also show enhanced sensitivity to WNV infection with high viral titers within the CNS [83]. Single TLR3 and TLR7 deficient mice displayed enhanced susceptibility to WNV infection, suggesting that MyD88 and TRIF mediated protection is the result of the engagement of multiple TLRs to fully protect and restrict virus dissemination within the CNS [84,85]. Along these lines, an essential role for TLR7 was shown during influenza A virus (IAV) and JEV CNS infection, where the lack of TLR7 signaling led to increased susceptibility and enhanced viral dissemination [84,85]. Interestingly, despite the enhanced susceptibility of MyD88^‒/‒^ mice to WNV infection, single TLR4 and TLR9 deficient mice show normal virus control, highlighting the redundancy of these systems and also suggesting that other components than TLR4 and 9 are critically involved in MyD88 dependent signaling during WNV infection [81,83]. In contrast, TLR4 deficient mice were highly susceptible to JEV infection and showed higher virus titers within the CNS than WT mice, which surprisingly was not associated with marked CNS inflammation [74]. Furthermore, synergistic signaling of TLR2 and TLR9 controls viral spread to the CNS compartment in models of HSV-2 infection that show virus kinetics and tissue spread similar to infected MyD88 deficient mice [86]. Arguably, the increased susceptibility of MyD88^‒/‒^ animals may be due to a deficiency in some critical innate or adaptive immune responses that functions in the CNS. Indeed, Sarangi et al. [87] reported impaired antigen-presentation function in MyD88^‒/‒^ mice during HSV-1 infection, which showed reduced inflammatory lesions and uncontrolled viral spread. TLR2 seems to play an ambiguous role, on one hand conferring protection by mitigating viral spread, and on the other hand by leading to potent inflammation that results in increased disease severity [86,87,88,89]. Deficiency of either TLR2 or TLR9 did not lead to induced encephalitis following intracorneal inoculation with HSV-1 [87]. However, dual deficiency appeared to diminish the protective effect orchestrated by these TLRs [88]. While TLR2 was shown to induce potent cytokine and chemokine responses, this did not necessarily confer protection, as illustrated by Kurt–Jones et al. [89], where HSV-1 infected mice lacking TLR2 showed protection from inflammatory lesions in the CNS which resulted in improved survival when compared with WT counterparts. During murine NiV infection, mice are naturally protected from lethal infection, whereas humans succumb to the infection in more than 50% of the cases [29,90]. This protection in the mouse model is mediated by both MyD88 and MAVS signaling, since single deficiencies for either MyD88 or MAVS did not affect survival upon NiV challenge, but the dual knockout rendered mice significantly more susceptible [91]. In mice lacking MyD88 and MAVS, viral loads increased in the peripheral organs and the brain while IFN-I transcripts decreased, suggesting compensatory mechanisms of TLR and RLR signaling in the control of NiV infection through IFN induction [91].

Collectively, TLR triggering and signaling via MyD88 contributes to a pro-inflammatory response in most of the investigated mouse models of viral encephalitis [80,81,87,89,91]. TLR engagement and MyD88 or TRIF mediated signal transduction resulted in protective effects by reducing viral loads in infection scenarios with VSV, WNV, IAV, JEV, HSV-1 or HSV-2, whereas it induced immunopathology in an HSV-1 infection model [80,81,82,83,84,85,86,87,91]. Some cellular PRRs have compensatory mechanisms during viral infection. For instance, different combinations of TLRs appear to compensate each other during the course of WNV infection [81,83]. Similarly, TLRs and RLRs seem to have redundant roles during NiV infection [91]. However, single TLRs may also function in a non-redundant manner, such as TLR4 during JEV infection and TLR7 during IAV and JEV infection [74,84,85]. While the precise mechanisms are very divergent in the different infection settings, the importance of TLRs during viral encephalitis is clearly underscored by these discoveries.

Whether neuropathological consequences are established during viral encephalitis rather through direct virus effects or by immunological reactions following TLR triggering remains only partially understood. The CNS is a highly sensitive organ and inflammatory processes are often a double-edged sword, controlling the virus as well as damaging the host [92]. Upon intranasal instillation with VSV, which is a highly cytopathic virus, infiltrating immune cells protect against lethal virus replication and viral dissemination within the CNS [80]. Notably in this process, CD8^+^ T cells were found to be indispensable [80,93]. Moreover, VSV’s neuroinvasive spread during peripheral infection was blocked by engaging TLR7 in subcapsular sinus macrophages in lymph nodes, highlighting the protective nature of the peripheral immune responses [94,95]. While in numerous neuroinfection models, virus clearance is supported by TLR activation, the activation of TLRs can also be the reason for the occurrence of inflammatory lesions during murine HSE, thereby increasing the disease’s severity [89]. Lymphocytic choriomeningitis virus (LCMV) infection portrays the variable possible outcomes of viral encephalitis of complete recovery, lethal acute encephalitis and chronic CNS inflammation as reviewed by McGavern [96]. Although LCMV is a non-cytopathic virus, severe damages can occur, which are mediated solely through the host’s immune response [97,98]. Especially, cytotoxic CD8^+^ T cells are highly activated and proliferative [99]. Interestingly, during in vitro LCMV infection, CNS glial cells mount pro-inflammatory cytokine responses via TLR2 and MyD88 [100]. Furthermore, MyD88 is required to induce activation and antiviral responses in CD8^+^ T cells [101]. In contrast to WT mice, MyD88^‒/‒^ mice do not show signs of weight loss and meningoencephalitis after intracerebral LCMV inoculation, while the phenotype detected in WT mice is mainly attributed to CD4^+^ T cell intrinsic TLR signaling [102]. In summary, these findings point towards a key role of TLR signaling in protective as well as harmful immune responses to viral CNS infection, while generally the protective effects outweigh the risk of causing damage in life-threatening viral infections [81,91].

## 3. Expression of Toll-like Receptors in Cells of the Central Nervous System

Global deficiencies of TLR family members and of components of their signaling pathways have shed light on the importance of TLR mediated sensing of neurotropic viruses, as well as the subsequent signaling through the adaptor molecules MyD88 and TRIF. The rediscovery of the meningeal lymphatic system terminated a long discussion about the existence of a CNS draining system [103]. Meningeal lymphatic vessels are found alongside the venous sinuses draining CNS parenchymal molecules into the cervical lymph nodes [104] with meningeal immunity taking a major role during CNS infection [105]. Upon virus entry into the brain parenchyma, CNS resident cells are productively infected, while several viral antigens are drained to lymph nodes, where potent immune responses are initiated. Thus, the resident cells of the CNS play a fundamental role in detecting invading pathogens and initiating innate immune responses. The major CNS resident cells are neurons, astrocytes and microglia, which express distinct combinations of TLRs and play different roles in virus sensing and the initiation of innate immune responses. Additionally, oligodendrocytes are involved in several immunological processes, notably through crosstalk with astrocytes and microglia [106,107]. However, due to the restricted repertoire of TLRs expressed in oligodendrocytes [108,109,110], this review will focus only on neurons, astrocytes and microglia. In vivo and in vitro studies aiming at understanding the impact of single TLRs within selected CNS-associated cell types are challenging due to technical limitations. Most of the studies investigating the role of TLRs during neurotropic virus infections deploy models where the impact of the adaptor molecules on the TLR signaling pathway, rather than single receptors, is investigated. Nevertheless, the expression of individual TLRs in neurons, astrocytes and microglia has been investigated in in vitro, in vivo, and ex vivo settings. While multiple studies highlighted the importance of TLRs in the context of viral encephalitis, the variable cellular expression of TLRs in the different types of CNS resident cells varies, as summarized in Table 1.

### 3.1. Neurons

Neurons are an essential, mostly non-renewable cell population constituting the main component of the nervous system. Multiple types of neurons have been described, such as sensory neurons that are essential to respond to stimuli, motor neurons that are involved in organization of movement and organ functions, and interneurons, which are crucial for maintenance of the neuronal network. Neurons have been shown to be the main target cells of zoonotic neurotropic viruses [147], as such viruses evolved strategies to target neuronal cells as part of their life cycle [148]. Considering the massive inflammation and the neuronal loss that often is associated with viral encephalitis, for long time it was assumed that infected neurons do not have the intrinsic capacity to mount innate immune responses [149]. However, more recently it became evident that neurons can also mount chemokine responses, which affect responses of neighboring and more distal cells [80,150]. In neurons, TLR signaling has been shown to be implicated in intrinsic regulation of neuronal functions such as morphology, morphogenesis and excitability. Since, during viral encephalitis, neurons often are the primary target of pathogens, more studies are needed to delineate the functional role of TLRs in the intrinsic neuronal biology during infection [149,151]. 

**Figure 1:**
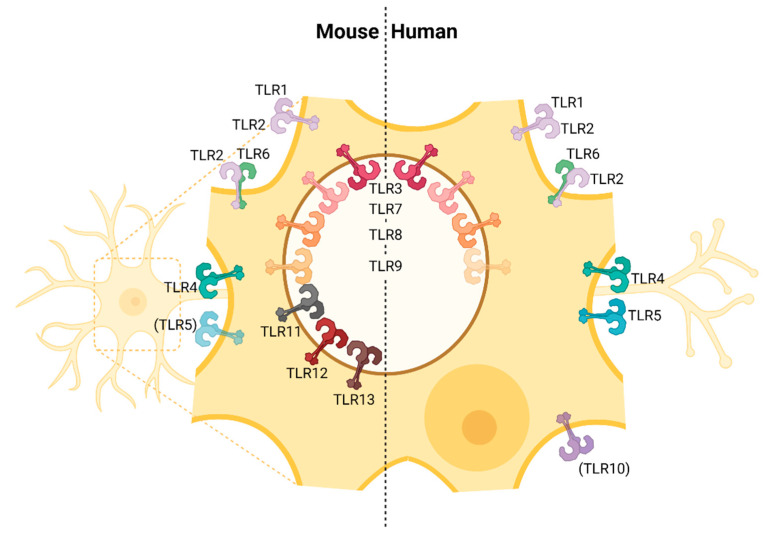
TLR expression in neurons from mice and humans. Virtually all TLRs known in the murine and human system are expressed in neurons. However, the expression of TLR5 in neurons from mice and TLR9 and TLR10 in neurons from humans are controversially discussed [70,111,113,114,118,122,123,125,126,127,128,129,130,137].

Analyses investigating TLR expression of neurons to some extent revealed conflicting results. Some studies reported that neurons do not express TLRs [152]. However, recent studies showed that across different species neurons do express a wide range of different TLRs [153]. Tang et al. [118] reported that murine neurons express TLR1–9 with TLR5 and 9 being expressed at particularly high levels, whereas TLR2 and 4 were intermediately expressed and TLR1, 3, 6 and 7 were the least expressed (Table 1, Figure 1). Zhou et al. [114] demonstrated that human neurons express all 10 human TLRs, although the expression level of single TLRs was variable across different neuronal cell subsets. Thus, currently available data indicate that neurons do express TLRs, and it is assumed that upon TLR stimulation neurons can mount innate immune responses.

Within infected neurons, the viral RNA is sensed through TLRs (and presumably other PRRs), which leads to the expression of cytokines such as IFN-β [151,152], TNF-α, IL-1β and IL-6 [154] and chemokines such as CCL2, CCL5, and CXCL10 [80,113,155]. While the direct involvement of neurons in leukocyte recruitment to the infected CNS has been debated for long time [156], recently, we could elucidate under in vivo conditions that upon intranasal VSV instillation, infected neurons are stimulated in a MyD88 dependent manner and then produce chemokines, which are critically involved in the recruitment of peripheral immune cells to the infected CNS [80].

During WNV infection, TLR signaling of neurons was shown to contribute to the inhibition of WNV replication, as the lack of MyD88 led to increased viral replication and decreased production of chemokines [81]. Similarly, in the case of Chikungunya virus infection, TLRs were proven to be essential in mouse neuronal cultures to mount pro-inflammatory cytokine responses and to inhibit viral replication [157]. TLR3 and TLR4 deficient primary cortical neurons mounted reduced IFN-I responses upon JEV infection and contained higher viral RNA copies than WT neurons [74]. Studies on human neuronal cultures showed an essential role of TLR3 in viral sensing and production of cytokines and chemokines [113,158]. Similar observations were made in murine neuronal cultures after WNV infection [73]. Recently, it was discovered that upon RABV and HSV-1 infection, human neurons express TLR3 and mount innate antiviral responses to dsRNA [158]. Interestingly, recently it was also shown that human iPSC-derived cortical neurons require functional TLR3 signaling to mount basal IFN-I responses [123]. Cortical neurons derived from a TLR3 deficient patient showed reduced tonic IFN-I expression, as opposed to iPSC-derived trigeminal neurons that lack constitutive TLR3-dependent IFN-I expression [123,159]. These data suggest that in TLR3 deficient patients, reduced tonic IFN-I expression results in reduced basal ISG levels within the CNS, which might enhance the vulnerability of such individuals to HSE.

### 3.2. Astrocytes

Astrocytes are the most abundant cell type of the CNS. They display functional heterogeneity and phenotypic plasticity depending on the effector cells present in their vicinity and the local milieu [160]. In the state of inflammation, including viral CNS infection, astrocytes undergo multifactorial and complex remodelling in response to pathological insults and become reactive, a process that often is referred to as astrogliosis [161]. Generally, the spectra of these heterogeneous changes vary with the aetiology and severity of CNS injury. Nonetheless, in the aftermath of astrogliosis several inflammatory and other immune mediators are induced. Indeed, we and others previously demonstrated that astrocytes predominantly mount protective IFN-I responses following brain infection with diverse neurotropic viruses [162,163,164]. Currently, astrocytes are considered as key contributors to the innate immune response of the CNS to infections and neurological disorders [165]. PRRs, which are essential for orchestrating innate and adaptive immune responses, are expressed by CNS cells including astrocytes [108,166]. These astrocytic PRRs are key danger sensors and facilitators of local neuroinflammation during CNS infection.

**Figure 2:**
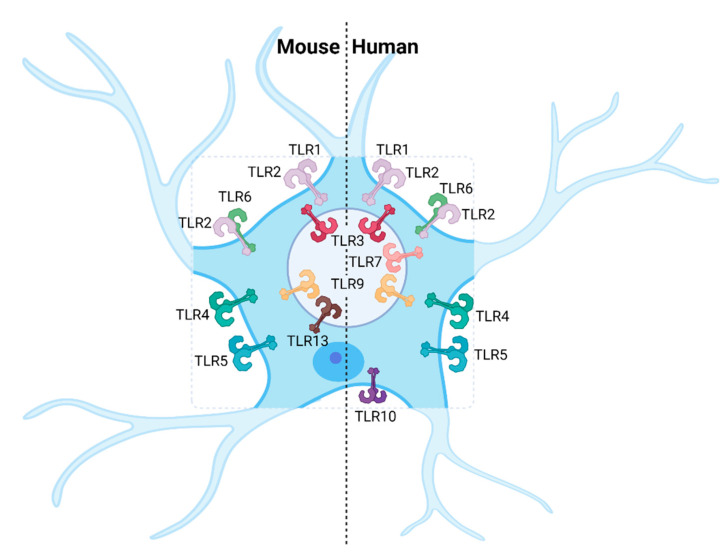
TLR expression in astrocytes. Most TLRs known are expressed in human and murine astrocytes, except for TLR8 in both systems, and TLR7, TLR11 and TLR12 in mice [70,108,111,112,115,120,121,125,126,137].

Astrocytes have been shown to express a distinct combination of TLRs (Table 1), although it is not clear yet whether some TLRs may change their expression during neuroinflammation. However, unlike microglia, astrocytes take longer to either upregulate TLRs or induce cytokines in response to TLR activation [167]. Expression of TLRs in human and murine astrocytes varies considerably. Human astrocytes express TLRs 1–7, TLR9 and TLR10 [108,115] while murine astrocytes express TLR1–6, TLR13 and, additionally, TLR9 upon inflammatory stimulation (Table 1, Figure 2) [70,111,112,120,121,125,126,137]. Importantly, under physiological conditions, both human and murine astrocytes predominantly express high levels of TLR3. Activation of the TLR pathway in astrocytes results in production of a wide range of neuroprotective and pro-inflammatory mediators, suggesting astrocytes are paramount for antiviral responses in the CNS.

The role of astrocytic TLRs in mounting innate immune responses to viral infection has been extensively studied in TLR deficient mice, which show increased susceptibility to various different infections [168]. Among all members of the TLR family, TLR3 is considered to be the front-line sentinel of viral infections and the primary mediator that induces immune responses against viruses since it recognizes dsRNA, an intermediate product of viral replication [34,169]. Unlike microglia, which express TLR3 intracellularly, astrocytes have both a high intracellular and cell surface expression of TLR3 [115]. This reflects the fact that astrocytes are no professional phagocytes and would require high extracellular expression of TLR3 for efficient detection of extracellular pathogens. Astrocytes devoid of TLR3 signaling show impaired type I IFN responses and increased permissiveness to HSV-2 infection in comparison to WT astrocytes [79]. Conditioned media from HSV-1 infected microglia selectively primed the TLR3 pathway of astrocytes, highlighting the crosstalk between glial cells during viral encephalitis [77]. Based on these findings, it is presumed that activation of astrocytic TLR3 controls dissemination of the virus. The TLR3 mediated astroglial response mounts neuroprotection to virally infected neurons and arrests virus spread to other CNS cells. Indeed, stimulation of organotypic human brain slices with the TLR3 agonist poly (I:C) significantly improved neuronal survival [166]. In vitro studies with primary murine astrocytes showed that HSV-1 infection induced TNF, IFN-β and multiple chemokines and inflammatory molecules via the TLR3 pathway [170]. 

Additionally, TLR3-dependent immune responses are also implicated in other infections with neurotropic viruses. Primary human astrocytes significantly increased expression of TLR3 and its downstream adaptor molecules, and thus promoted the release of pro-inflammatory cytokines and chemokines following ZIKV infection. Pharmacological inhibition of TLR3 signaling decreased these inflammatory responses, highlighting the role of TLR3 in ZIKV infection [171]. Moreover, in a recent transcriptomic study, we observed significant upregulation of TLR3 and subsequent induction of anti-viral responses in murine primary astrocytes upon TBEV infection [172].

In contrast, Carpentier et al. [173] reported that the TLR3 mediated pro-inflammatory response in astrocytes is dispensable following infection with Theiler’s murine encephalomyelitis virus (TMEV). Indeed, astrocytes devoid of TLR3 showed reduced pro-inflammatory responses upon TMEV infection, but such reduced responses did not affect in vitro virus replication in astrocytes [174]. Instead, it appeared that TMEV-infected astrocytes heavily relied on intracellular protein kinase R (PKR) activation for induction of antiviral responses. Collectively, the production of chemokines in brain parenchyma following virus infection could potentially act as a signal for recruitment of pathogen-specific CD4^+^ and CD8^+^ T cells. However, it is crucial to investigate the spatiotemporal activation of TLR3 signaling that may have differential immunopathological outcomes [75,173,174]. 

TLR2 is known to sense microbial ligands. However, it has been demonstrated to mediate the production of pro-inflammatory cytokines in response to HSV-1 [89] and vaccinia virus [175]. In the context of CNS infection, TLR2^‒/‒^ primary astrocytes showed lower TMEV-induced NF-κB activity than WT cells [176]. These experiments demonstrated that TLR2 is crucial for NF-κB activation, leading to downstream cellular activation and cytokine production in astrocytes following TMEV infection. Interestingly, TLR2 expression in TLR3^‒/‒^ astrocytes was dramatically lower than in in WT astrocytes. Therefore, it is likely that TLR3 mediated signaling in astrocytes is a pre-requisite for TLR2 expression and subsequent TLR2-dependent induction of pro-inflammatory cytokines, such as IL-6 and IL-1β in the CNS. Such amplification of cytokines favors the more pathogenic subsets of T cells such as Th17 [177]. 

Unlike microglia, the expression of TLR4 in astrocytes is debated. Several groups have been unable to demonstrate TLR4 expression in astrocytes, both in vitro [165] and in vivo [178]. Nevertheless, others have shown low and constitutive astrocytic TLR4 expression that seems to increase upon cell activation [108,179]. Such inconsistencies could be due to differences in in vitro versus in vivo experimental setups and TLR assay detection limits, as well as in the purity of astrocyte primary cultures. Nevertheless, TLR4 seems to be involved in the sensing of HSV-1, even though the mechanism is still only partially elucidated [180]. The upregulation of TLR4 in murine astrocyte cultures during productive, and not during abortive, HSV-1 infection has been observed, as well as IRF3 phosphorylation and an increased expression of IRF7 [180]. One proposed possibility of TLR4 activation during viral infection is the appearance of endogenous danger signals triggering TLR4, and indeed the danger signal acute phase protein 3 was heavily upregulated in productively infected astrocytes [180].

Astrocytes also express TLR9, a receptor for unmethylated DNA with CpG motifs. Stimulation of astrocytes with CpG ODN induced p38 MAPK activation and subsequent expression of inducible nitric oxide synthase (iNOS) in a MyD88-dependent manner [181]. Other studies have reported TLR9 agonists to induce astrocyte-derived chemoattractants [182]. Recently, Hamel et al. [183] reported a significant increase in TLR7 and TLR9 expression in ZIKV infected primary human astrocytes. However, it still remains elusive whether increased astroglial TLR7 and TLR9 expression contributes to antiviral responses of astrocytes against ZIKV infection. Astrocytes have also been reported to express TLR1, TLR5, TLR6 and TLR8 [108], however, the immune functional role of these TLRs has not been investigated in vivo in the context of virus infection.

### 3.3. Microglia

Microglia arise from erythromyeloid progenitors of the yolk sac that seed the CNS parenchyma during embryonic development, and develop into tissue resident macrophages of the CNS [184,185]. Postmortem brain analysis of individuals who succumbed to lethal viral encephalitis revealed pronounced myeloid cell activation [186,187]. In the murine system, microglia are of pivotal significance for protection against CNS virus infection. Pharmacological depletion of microglia or knockout mice with reduced abundance of microglia showed increased sensitivity to viral challenges [188,189,190,191,192,193]. 

**Figure 3:**
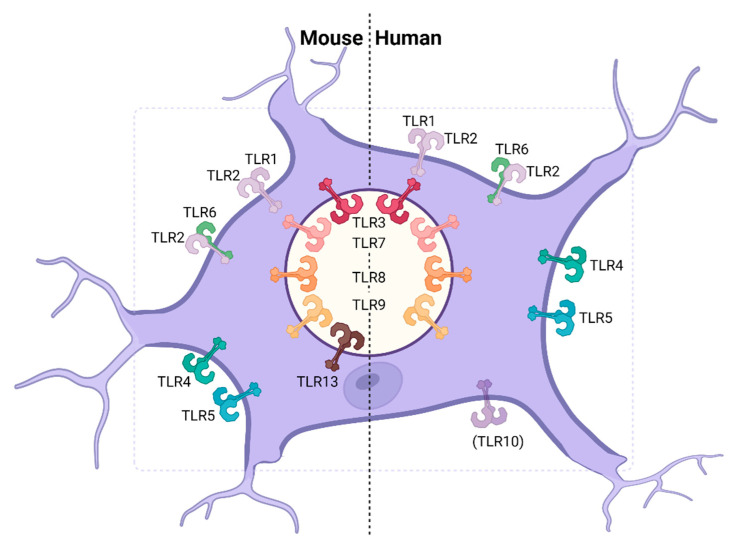
TLR expression in microglia. Human and murine microglia express a high number of TLRs, whereas TLR11 and TLR12 were not detected in the murine system and the expression of TLR10 remains to be defined [70,108,111,115,121,122,125,126,131,137].

Microglia have been described to respond in vitro and in vivo to TLR agonists, suggesting that they do express a broad repertoire of functional TLRs in the murine system and in humans. In mice, TLR1–5 and 7–9, as well as low levels of TLR13, have been described to be expressed in microglia (Table 1, Figure 3) [70,111,121,122,125,126,131,137]. In humans, expression of TLR1–9 have been reported, while current data is inconsistent on the expression of TLR10 in human microglia (Table 1, Figure 3) [108,115]. TLR10 appears to be downregulated during aging, which might be the reason for divergent findings [135]. Differences in TLR expression of microglia described in different studies might be attributed to the background of the mouse strains analyzed [115,194,195]. Histological analyses of brains from WNV-infected mice revealed that only a minor percentage of microglia was co-labeled with the WNV envelope protein (WNV-E) and that TLR3 deficiency did not affect the permissiveness of the cells [73]. Furthermore, WNV infection of primary mouse microglia led to an increase in TLR3 expression, whereas TLR3^‒/‒^ microglia showed complete shut-off of IL-6 and TNF-α expression when compared with WT microglia [71]. Similarly, knocking out TLR3 by RNA interference (RNAi) resulted in decreased TNF-α, IL-6 and CCL2 responses following JEV infection of the BV2 murine microglia cell line [196]. In accordance with these findings, TLR3 deletion improved protection from virus induced neuroinflammation by reducing microglial activation [71]. Moreover, upon in vitro infection of BV2 cells with DENV, pharmacological inhibition of TLR3 signaling abrogated microglia migration in wound healing assays [197]. TLR3 deficiency of primary microglia leads to a partial impairment of IFN-I responses upon HSV-1 infection, suggesting that the cGAS-STING pathway is the predominant pathway triggering IFN-I responses in these cells in vitro [70,77]. During HSV-1 skin infection, TLR3 and TRIF are required for efficient priming of gB-specific CD8^+^ T cells by cross-presenting DCs [198]. Although these experiments selectively focused on HSV-1 lesions of the skin, it cannot be excluded that cross-presenting microglia require functional TLR3 signaling to coordinate CD8^+^ T cell function within the brain during HSE. Moreover, microglial TLR7-dependent in vivo sensing of the virus drives homing of the infiltrating cells towards WNV infected cells through IL-23 production [199]. Furthermore, several cytokines and chemokines, such as IFN-β, IL-6, TNF-α and CXCL10, have been shown to be regulated by TLR2 signaling upon HSV-1 infection in primary murine microglia [200]. Specifically, TLR2 binds the HSV-1 glycoproteins gB and gH/gL. Even in their soluble form, gB and gH/gL are able to trigger TLR2 and subsequently activate NF-κB [46]. Conversely, TLR4 might play a dual role in the sensing of neurotropic viruses. In astrocytes, as previously illustrated, TLR4 might sense virus-induced cellular danger signals [180]. On the other hand, intracerebroventricular injection of neuraminidase, which can be found on the surface of viral and bacterial particles, led to microglial proliferation and IL-1β, IL-6 and TNF-α induction, implying direct sensing of viral and bacterial PAMPs [201]. Moreover, expression of the HSV-2 immediate early protein ICP0 in an epithelial cell line led to the upregulation of TLR4 and the phosphorylation of the transcription factor AP-1, whereas it is still unclear whether this mechanism applies also to microglial cells [202]. Nevertheless, these observations suggest that TLR4 might play an important role in virus sensing during neurotropic viral infections, possibly through direct sensing of viral proteins through microglia and indirect sensing of danger signals via astrocytes. Interestingly, the cellular E3 ubiquitin ligase Peli1, which is highly expressed in microglia, directly regulates TLR mediated cytokine responses by TRAF3 degradation [203]. During WNV infection, Peli1^‒/‒^ primary murine and human microglia showed reduced viral replication capacity and cytokine secretion compared to WT cells [204]. 

## 4. Immune Evasion of the TLR Pathway by Neurotropic Viruses

Following viral infection, nucleated cells sense virus particles and viral structural components through PRRs, such as TLRs, and initiate an innate immune response to inhibit viral replication and dissemination. Notably, the IFN response and IFNAR signaling leading to ISG induction play a central role in viral restriction, as many viruses are sensitive to IFNs and ISGs. Correspondingly, many viruses evolved strategies to evade the sensing or to interfere with the underlying signaling cascades. Almost every step of the TLR signaling cascade is targeted by different viruses. Such elaborate viral countermeasures include degradation of TLR signaling components, disruption of formation of signaling complexes, interference with activity of transcriptional factors, deubiquitination of signaling molecules, and molecular mimicry of cellular proteins. In the following, we highlight few examples of how viruses exploit some of these strategies.

### 4.1. Herpes Simplex Virus 1

HSV-1 derived nucleic acids and viral proteins can be detected via TLR2, 3, 4, 7 and 9 [86,170,180,205]. Specifically, TLR2 binds the HSV-1 glycoproteins gB and gH/gL [46], while the dsRNA sensor TLR3 can be triggered by dsRNA intermediates, which are produced during the HSV-1 replication cycle [169,206]. TLR4 is significantly involved in HSV-1 sensing as well, even though the mechanism still remains to be elucidated [180]. TLR7, recognizing ssRNA, and TLR9, binding unmethylated CpG DNA, contribute to the initiation of innate immune responses during HSV-1 infection [86,205]. Despite offering a wide range of TLR ligands, HSV-1 establishes lifelong latency in neuronal cells [207]. To counteract elimination through innate immune activation, HSV-1 encodes proteins that interrupt the intracellular signaling cascade following TLR triggering, as recently reviewed by Zhu et al. [208]. Along these lines, TLR2 mediated signaling can be abrogated by the viral protein ICP0 through the proteasomal degradation of MyD88 [209]. Furthermore, the HSV-1 encoded US3 protein kinase inhibits the agglomeration of the master transcription factor NF-κB in the nucleus, thus hindering the transcriptional activation of pro-inflammatory factors as well as impeding the upregulation of TLR3 [210,211]. The HSV-1 tegument protein VP16 interferes with TLR signaling by repressing the binding of IRF3 to its coactivator CREB binding protein (CBP) that is crucial for the transcriptional induction of IFN-I and other cytokines [212]. 

### 4.2. Flaviviruses

Several members of the Flaviviridae gain entry into the CNS, leading to severe neurological disease. The patients’ immune response is of critical relevance to control such virus infections, as demonstrated by a series of organ transplanted patients under immunosuppression who developed severe and mostly fatal WNV infection [213]. WNV and other neurotropic flaviviruses share a similar genetic background for their structural and nonstructural proteins (NS). DENV, WNV, TBEV and ZIKV contain homologues of the NS1, NS2A, NS2B, NS3, NS4A, NS4B and NS5 with overlapping functions. Among these potentially neurotropic flaviviruses, host sensing of DENV and its evasion mechanisms are well documented [214]. DENV can be detected through TLR3, 7 and 8, while TLR3 seems to be of particular relevance [215]. Multiple DENV NSs antagonize the induction of IFN-I as well as the IFN-I signaling, although to date, no TLR signaling specific mechanism has been elucidated. Instead, the cGAS-STING mediated IFN-I induction is targeted through cleavage of STING by the NS2B/3 complex [214,216]. Downstream of IFNAR triggering, DENV NS4A and NS4B cluster around STAT1, thus sequestering it from being activated and translocated to the nucleus [217]. DENV, as well as WNV, produce a series of noncoding subgenomic flavivirus RNAs (sfRNAs). These sfRNAs primarily antagonize the host’s RNAi system, whereas the WNV sfRNA has additional effects, by neutralizing the antiviral effect of IFN-I [218]. Furthermore, WNV also offers a repertoire of proteins counteracting TLR signaling. The WNV-E is a robust inhibitor of dsRNA mediated IFN-I and ISG induction. Upon dsRNA treatment, WNV-E prevents the polyubiquitination of receptor-interacting protein 1 (RIP1) required for NF-κB activation [219]. Finally, the TBEV related Langat virus (LGTV) constricts IFN-I signaling by inhibiting and, therefore, delaying STAT1 phosphorylation through activity of NS5 [220,221]. 

### 4.3. Rabies Virus

RABV of the Rhabdoviridae family confers an extraordinarily fast and detrimental disease course, and since RABV travels between neuronal cells, its recognition by neurons is especially critical. Indeed, neurons have been reported to mount IFN-I responses and upregulate a broad range of immune-related genes upon RABV infection, possibly mediated by TLR3 sensing [158]. However, RABV contains multiple proteins with IFN-I antagonistic effects [222]. With that respect, the RABV P protein acts on TBK1, which results in reduced IRF3/7 phosphorylation [223]. Comparable to the LGTV NS5, the RABV P protein also inhibits STAT1 phosphorylation following IFNAR triggering, which is crucial to protect the RABV progeny from IFN-I mediated antiviral effects [224]. The RABV matrix (M) protein interacts directly with RelAp43, which in turn regulates NF-κB mediated transcriptional activation [225]. Moreover, the rhabdoviral N protein covers the genomic RNA as well as the antigenome produced during the replication cycle, possibly impairing recognition by PRRs [226]. 

### 4.4. Influenza a Virus

IAV is primarily a pulmonary pathogen belonging to the Orthomyxoviridae family, but can reach the CNS and cause acute encephalitis or encephalopathy [227,228]. Similar to the rhabdoviral N protein, the IAV NS1 coats dsRNA, and thus hides the viral genome from detection through PRRs [229].

Collectively, these data demonstrate how TLRs are directly involved in sensing viral components [86,170,180,205] and through which evasion strategies of TLR signaling virus sensing is impaired by neurotropic viruses such as HSV-1, DENV, WNV, RABV [214,215,216,217,218,219,220,221,222,223,224,225]. However, the binding and recognition of viral proteins by TLRs is just on the verge of being understood. Thus, more studies are needed to fully elucidate the physical interactions, which is the basis for exploitation of these mechanisms as immunotherapeutic treatments during viral encephalitis or as potential adjuvants during vaccination.

## 5. Primary Defects in the TLR3-IFNAR Axis Can Affect the Clinical Outcome of Viral Encephalitis

Several genetic variations in components of the TLR3–IFNAR signaling cascade have been associated with cases of childhood HSE during primary infection [69,230]. The first primary immunodeficiency that was connected with HSE was a homozygous two-nucleotide deletion in STAT1 in one infant that eventually succumbed to the infection [231]. Recently, a homozygous large deletion in IFNAR1 was identified in a child with lethal HSE clinical outcome, which resulted in a truncated form of IFNAR1 that was unable to bind to TYK2, thus causing IFN-I unresponsiveness [232]. 

The importance of TLR3 signaling during HSE was uncovered upon the discovery of UNC-93b. Initially, a broad chemically induced mouse germline mutagenesis screen revealed the importance of UNC-93b in regulating endosomal TLR-mediated cytokine responses [233]. Coincidentally, the observation that peripheral blood mononuclear cells (PBMCs) and fibroblasts isolated from two HSE patients showed diminished IFN-I responses upon exposure to viruses and endosomal TLR agonists, when compared with cells from healthy individuals led to the identification of two distinct autosomal recessive (AR) variants in the UNC93B1 allele [234]. Nowadays, it is well documented that UNC-93b is a chaperone directly controlling the stability and trafficking of endosomal TLRs [235,236,237]. The second genetic etiology for HSE came with the identification of an autosomal dominant (AD) nucleotide substitution in TLR3 in two unrelated children with HSE, that resulted in an amino acid exchange within the TLR3 region that is essential for dsRNA binding [51]. Surprisingly, HSV-1 seropositive relatives who also were heterozygous for that substitution did not have clinical history of HSE, indicating that this AD variant may show incomplete clinical penetration. Similarly, incomplete clinical penetration was later observed in almost all HSE inborn errors. Furthermore, the observation that PBMCs and several leukocyte subsets from an HSE patient with two compound heterozygous complete loss-of-function TLR3 variants responded normally to poly (I:C) and HSV-1 infection, as opposed to fibroblasts, indicated that TLR3 signaling may be redundant in peripheral immune cells, but not within the CNS compartment [238]. The definitive answer to that came with the establishment of the induced pluripotent stem cells (iPSC) technology. With this technology, dermal fibroblasts from HSE patients with UNC-93b and TLR3 deficiencies were reprogrammed to neural stem cells, neurons, astrocytes and oligodendrocytes [239]. Upon HSV-1 exposure, iPSC-derived neurons and oligodendrocytes from UNC-93b deficient patients were highly susceptible to HSV-1 infection, whereas control cells did not show this effect, indicating for the first time that TLR3 deficiencies control CNS intrinsic anti-HSV-1 immunity [239]. AR nonsense and AD missense mutations in TRIF in two unrelated HSE patients further highlighted the importance of TLR3 signaling for the development of HSE [240]. Notwithstanding, the identification of AD variants in TRAF3, TBK1 and IRF3, which are downstream mediators of several PRR signaling pathways, in patients suffering from HSE broadened the knowledge even more concerning HSE-related inborn errors [241,242,243]. Recently, a heterozygous missense mutation in TLR3 was identified in a patient suffering from VZV encephalitis, although in that study the variant was not tested for functional defects [244]. 

Although remarkable knowledge has been gained from HSE patients with inborn defects in the TLR3 pathway, in the recent past, TLR3-pathway risk alleles have been implicated in underlying pathogenesis of tick-borne encephalitis (TBE). Genetic screenings have identified the TLR3 rs3775291 allele as a risk factor in TBEV infection [245]. TLR3 rs3775291 is a non-synonymous mutation (G > A, Leu412Phe) leading to impaired receptor function. In vitro studies on HEK293 cells transfected with Phe-412 constructs have shown abrogated TLR3-signaling [246]. Similarly, such mutants have shown reduced Coxsackie virus mediated TLR3 signaling and increased viral replication, suggesting induction of a reduced inflammatory response. Consequentially, the overall outcome of the TLR3 rs3775291 mutation is reduced induction of NF-κB and IFN signaling in homozygous individuals, who also show poor immune responses against several pathogens [247] and likely have an increased risk of developing TBE. Moreover, Ishizaki et al. [248] reported an association between the TLR3 rs3775291 mutation and an increased risk of developing sclerosing panencephalitis caused by measles virus. However, more functional assays are required to investigate the link between TLR3 gene defects and cases of viral encephalitis.

Genetic variations in TLR3-mediated immune responses can as well explain severe complications manifested in some influenza patients. Genetic analysis on all TLRs involved in the recognition of IAV in patients with influenza-associated encephalopathy documented a missense mutation (F303S) in the TLR3 locus [249]. A single point mutation (T > C) caused amino acid transition from phenylalanine to serine at position 303. HEK293 cells transfected with the mutant showed reduced IFN-β production and diminished NF-κB activity when compared to WT cells. Although additional studies are needed to further clarify this association, the available data point towards F303S being a loss-of-function mutation of TLR3.

## 6. Innovative TLR Agonist Mediated Intervention Strategies and Enhancement of Vaccine Responses against Neurotropic Virus Infections

The triggering of innate immune sensors for the treatment of communicable as well as non-communicable diseases is intensively investigated, also in the context of clinical application. This is true for innovative cancer treatments [250], and for the development of new adjuvants, i.e., enhancers of vaccination-induced immune responses [251,252]. Despite the availability of vast quantities of promising experimental data, to date, only the TLR4 ligand monophosphoryl lipid A and TLR7 triggering imiquimod are used in clinics [253]. Both small molecules are used for the treatment of cancer, but potentially could also be exploited for the treatment of infectious diseases and, in particular, of viral encephalitis. This is true because TLR4 and TLR7 have been reported to contribute to the sensing of viruses and to the initiation of innate immune responses against neurotropic viruses such as HSV-1, DENV, WNV and RABV [158,199,215]. 

As demonstrated by individuals with inborn defects in TLR signaling and by experimental models, the immune response against viruses is of key relevance to control neurotropic viral infections. In reality, however, viruses often evade innate immune responses and cause neurological symptoms, which often have lethal outcomes. The additional enhancement or stimulation of the innate immune sensing in the CNS is therefore a promising strategy for the treatment of viral encephalitis.

Sato et al. [70] have described the mechanism by which TLR3 senses HSV-1 in neurons and astrocytes and subsequently activates the mammalian target of rapamycin (mTOR) complexes (mTORC), resulting in the induction of IFN-I responses that trigger antiviral effects. mTOR is a central regulator of various metabolic processes including glycolysis, fatty acid and cholesterol metabolism and the tricarboxylic acid (TCA) cycle. Moreover, the anabolic events of mitochondrial biogenesis and synthesis of the endoplasmatic reticulum (ER) and the Golgi apparatus are controlled by mTOR [254]. In detail, poly (I:C)-triggered TLR3 was found to bind to mTORC2 along with TRAF6 in mouse embryonic fibroblasts (MEFs). Upon HSV-1 infection, TRAF3 co-precipitated with components of mTORC1, suggesting the presence of a TLR3—mTORC2—TRAF—mTORC1 signaling cascade. The inhibition of mTORC1/2 led to decreased survival and higher viral loads in a TLR3-dependent manner. Additional triggering of the TLR3–mTOR axis by applying agonistic anti-TLR3 mAbs significantly improved survival of HSV-1 infected mice and increased Ifnb1 and Ccl5 expression in vivo as well as in vitro in primary murine neuronal cultures, while viral loads were reduced. Interestingly, the protective effect of TLR3 disappeared when treated with the mTOR inhibitor Torin1 [70]. Applications of mAbs have been increasingly used in the clinics for the treatment of a variety of diseases, such as cancer, autoimmune conditions, and infectious diseases [255]. Notably, in the context of the causative agent of the coronavirus disease 2019 (COVID-19), i.e., the severe acute respiratory syndrome coronavirus 2 (SARS-CoV-2), several mAbs have been successfully applied in risk patients. The use of mAbs targeting TLRs and other PRRs thus offers attractive options for the treatment of viral encephalitis.

Several vaccines against neurotropic viruses are licensed and broadly applied in clinical use. Fatal disease courses due to Japanese encephalitis and rabies are almost completely vaccine preventable with a vaccine effectiveness of 60–97% for JEV and up to 100% for RABV infection [256,257,258,259,260,261]. On the other hand, the vaccines currently available against TBEV are not completely efficient against all virus strains, partly due to rather low immunogenicity of the virus [262,263]. TLR agonists might be suitable adjuvants in order to increase immunogenicity of existing or novel TBEV vaccines. To date, no vaccines are licensed for human use against WNV, DENV or HSV-1. A number of WNV vaccine candidates that are based on WNV-E or inactivated viral particles have entered phase I or phase II human clinical trials. Similar to the currently available TBEV vaccines, some WNV vaccines have only demonstrated modest immunogenicity [264]. Van Hoeven et al. [265] propose the enhancement of immunogenicity of WNV vaccine platforms by the addition of the TLR4 agonist synthetic lipid-A formulated in a stable oil-in-water emulsion. This study in mice showed enhanced activation of Th1 CD4^+^ T cells, and an expansion of long-lived antibody secreting cells, which was associated with the induction of higher WNV neutralizing antibody titers. 

In 2020, mRNA-based vaccines were licensed for the first time. The vaccines against COVID-19 developed by BioNTech/Pfizer and Moderna display impressive immunogenicity and efficacy in clinical trials [266,267]. Despite SARS-CoV-2 being a primarily respiratory pathogen, viral antigens were also found in the brain parenchyma and in single cases in the cerebrospinal fluid (CSF) of severely affected COVID-19 patients [268,269,270]. Song et al. [271] demonstrated neuroinvasion of SARS-CoV-2 in human brain organoids, ACE2-expressing mice, and in human post-mortem tissues. Despite neurological symptoms that are associated with COVID-19, as well as long COVID-19, SARS-CoV-2 mostly is a respiratory pathogen and only rarely causes neurotropic infections. Correspondingly, COVID-19 can be considered a multi-organ disease [272]. Nevertheless, the currently available vaccines prevent severe disease courses and hospitalization, hence they are also protecting against serious neurologic manifestations. A new generation of an RNA-based RABV vaccine candidate has previously been developed and characterized in a preclinical setting by Stitz et al. [273]. Here, intradermal administration of mRNA of the RABV glycoprotein in mice induced robust neutralizing antibody titers and conferred protection against RABV lethal challenge. Interestingly, these mRNA-based vaccines do not require the addition of adjuvants [266,267,273]. Non-self mRNA molecules thus have an intrinsic immunostimulatory potential and can be used as adjuvants, as illustrated by Ziegler et al. [252]. Synthetic ssRNA RNAdjuvant enhanced antibody responses in mice upon immunization with the IAV subunit vaccine Influvac. Moreover, dendritic cells were activated in a TRL7 and MyD88 dependent manner upon in vivo stimulation with RNAdjuvant only, while the RLR sensing pathway granted an additional immunoenhancing influence in the co-immunization setting [252]. Since mRNA can be sensed through RLRs, TLR3, TLR7 and TLR8 [38,274], and those TLRs are prominently expressed in CNS resident cells, RNA-based vaccines are promising candidates for the prevention of viral CNS infection.

Inspired by efficacious vaccines that are based on complete microbial particles, which comprise multiple TLR ligands, such as the live attenuated yellow fever virus vaccine, the application of multi-TLR agonists was proposed. Concomitant stimulation of TLR2 and TLR7 of CD4^+^ T cells that were latently infected with human immunodeficiency virus 1 (HIV-1) caused viral reactivation and subsequent induction of antiviral factors [275]. This mechanism could be exploited for the treatment of patients latently infected with HSV-1, VZV or HCMV, prior to a generalized immunosuppression e.g., in the case of organ transplantation, which has the risk of virus reactivation and development of an encephalitis. A similar approach was followed by Hensel et al. [276] with the design of an HSV-2 vaccine. HSV-2 is a close relative of HSV-1 and causes predominantly genital herpes, whereas both HSV-1 and HSV-2 can cause neonatal herpes. The disease is associated with high morbidity and mortality manifesting at the level of the skin, eyes and mouth, the CNS or as a systemic disease. Therefore, a vaccine against HSV-1 and HSV-2 is needed as a preventive measure against herpes viral CNS infections in adults as well as newborns. Hensel et al. [277] combined HSV-2 antigens with different TLR agonists in the prospect of enhancing the immune reaction. Indeed, the joint stimulation of the HSV-2 antigens along with ODN 2395 (TLR9 ligand) or poly (I:C) (TLR3 ligand) or MPL (TLR4 ligand) resulted in the induction of robust cytokine responses and a dramatically increased HSV-2 specific killing capacity of murine splenocytes. Moreover, a vigorous antibody response was initiated, and the disease score was drastically reduced with lower copy numbers of latent HSV-2 [276]. The use of poly (I:C) was furthermore employed for the treatment of HeV infected HeLa cells, which resulted in reduced numbers of viral particles [278,279]. This is an interesting finding pointing towards further investigation of endothelial cells, especially those constituting the blood vessels around the CNS.

Although virus recognition by TLR initiates many intrinsic antiviral defenses, TLR stimulation can enhance disease severity, as exemplified by TLR3 and TLR4-mediated detrimental inflammatory responses during influenza and WNV infection [71,280,281,282]. These alarming findings necessitate the development of therapeutics that counteract these TLRs and thus alleviate virally induced disease symptoms. Driven by this concern, Shirey et al. [283] demonstrated that the application of the TLR4 antagonist Eritoran abrogated IAV-induced lethality in mice, which correlated with the inhibition on virus-induced inflammatory responses.

In summary, a broad range of TLR agonists with immunomodulatory properties offers infinite possibilities to affect antiviral responses during acute viral encephalitis and upon vaccination against neurotropic viruses, which remain be fully exploited.

## 7. Conclusions

TLR-mediated pathogen sensing plays a key role in the pathogenesis of viral encephalitis. Extensive data from human patients with inborn genetic defects, as well as studies in mice, highlight a central role of TLR3 during neurotropic virus infections [69,73,123,238,239]. Furthermore, TLR2, TLR4, TLR7, TLR8 and TLR9 take part in the initiation of the immune response against neurotropic viruses [85,86,89,201,282]. CNS resident cell subsets including neurons, astrocytes and microglia express distinct patterns of TLRs, whereas the overall expression levels vary between the different cell subsets [70,108,111,112,113,114,115,118,120,121,122,123,125,126,127,128,129,130,131,137]. Thanks to the increasing number of studies about the exploitation of TLR-mediated sensing and TLR signaling, important knowledge is being collected about the potential of preventive and therapeutic interventions against neurotropic infections [70,158,199,215,283,284]. Novel cutting-edge technologies, such as single cell RNA sequencing, will further contribute to unraveling the mechanisms relevant for TLR mediated virus sensing in a more precise manner on the cellular level. Thus, targeting TLR signaling cascades is a promising approach to develop new intervention strategies for the therapy of viral encephalitis.

## Figures and Tables

**Table 1:** TLR expression in CNS resident cell subsets. CNS: Central nervous system, DRG: Dorsal root ganglia, EP: Envelope protein, FC: Flow cytometry, GP: Glycoprotein, hiPSC: Human induced pluripotent stem cell, ICC: Immunocytochemistry, IF: Immunofluorescence, IR: Immunoreactivity, LTA: Lipoteichoic acid, LPS: Lipopolysaccharide, PGN: Peptidoglycan, RNAseq: RNA sequencing, RT-PCR: Reverse transcription PCR, WB: Western blot. +: Expressed, −: Not expressed.

**Receptor**	**Ligand**	**Expression on CNS-Resident Cells**					
		**Mouse**			**Human**		
		**Neurons**	**Astrocytes**	**Microglia**	**Neurons**	**Astrocytes**	**Microglia**
TLR1 [41]	Bacterial lipo-proteins [41]	+ IF (in vivo) [111]	+ RT-PCR (in vitro, primary astrocytes), IF (in vivo) [111,112]	+ IF (in vivo) [111]	+ RT-PCR (in vitro, cell line) [113,114]	+ (low) RT-PCR (in vitro, primary astrocytes) [115]	+ RT-PCR (in vitro, primary microglia) [108,115]
TLR2 [35]	PGN, LTA [116,117], viral EPs, GPs, core proteins	+ IF (in vivo), IF (in vitro, primary cortical neurons) [118,119]	+ RT-PCR, FC, WB (in vitro, primary astrocytes), FC (ex vivo) [112,120,121]	+ RT-PCR (in vitro, cell line), FC (ex vivo) [121,122]	+ RT-PCR (in vitro, cell lines) [113,114,122]	+ (low) RT-PCR (in vitro, primary astrocytes) [108,115]	+ RT-PCR, IF (in vitro, primary microglia) [108,115]
TLR3 [34]	dsRNA, poly(I:C) [34]	+ FC (in vitro, primary neurons) [70]	+ RT-PCR, FC (in vitro, primary astrocytes) [70,112]	+ RT-PCR (in vitro, cell line), FC (in vitro, primary microglia) [70,122]	+ RT-PCR (in vitro, primary neurons and cell lines), WB (in vitro, hiPSC neurons) [113,114,122,123]	+ RT-PCR, FC (in vitro, primary astrocytes) [108,115]	+ (low) RT-PCR, FC (in vitro, primary microglia) [108,115]
TLR4 [33,124]	LPS [33,124], GPs, EPs, fusion proteins [47,48]	+ RT-PCR (in vitro, primary neurons) [125]	+ RT-PCR, FC (in vitro, primary astrocytes) [112,120,125]	+ RT-PCR, WB (in vitro, cell line), RT-PCR (in vitro, primary microglia) [122,125]	+ RT-PCR, WB (in vitro, cell lines and primary microglia) [113,114,122]	+ (low) RT-PCR (in vitro, primary astrocytes) [115]	+ RT-PCR, IF (in vitro, primary microglia) [108,115]
TLR5 [37]	Flagellin, profilin [37]	− IF (in vitro, primary neurons) [126] + microarray (in vitro, primary cortical neurons) [118]	+ RT-PCR, IF (in vitro, primary astrocytes) [112,120,126]	+ IF (in vitro, primary microglia) [126]	+ RT-PCR (in vitro, primary neurons) [114]	+ (low) RT-PCR (in vitro, primary astrocytes) [115]	+ (low) RT-PCR (in vitro, primary microglia) [108,115]
TLR6 [40]	Diacyl lipo-peptides [40]	+ IF (in vitro, primary DRG neurons) [127]	+ RT-PCR (in vitro, primary astrocytes), FC (ex vivo) [112,121]	− FC (ex vivo) [121]	+ RT-PCR (in vitro, primary neurons and cell lines ) [114]	+ (low) RT-PCR (in vitro, primary astrocytes) [115]	+ (low) RT-PCR (in vitro, primary microglia) [108,115]
TLR7 [36,38]	ssRNA [36,38]	+ WB, RT-PCR (in vitro, primary DRG and CNS neurons) [128,129]	− RT-PCR (in vitro, primary astrocytes) [112]	+ RT-PCR (in vitro, cell line) [122]	+ (low) RT-PCR (in vitro, cell lines and primary neurons) [114,122]	+ (low) RT-PCR (in vitro, primary astrocytes) [115]	+ RT-PCR (in vitro, primary microglia) [108,115]
TLR8 [38]	ssRNA [38]	+ WB, RT-PCR (in vitro, primary DRG and CNS neurons) [128,129]	− RT-PCR (in vitro, primary astrocytes) [112]	+ RT-PCR (in vitro, cell line) [122]	+ (low) RT-PCR (in vitro, cell lines and primary neurons) [114,122]	− RT-PCR (in vitro, primary astrocytes) [115]	+ (low) RT-PCR (in vitro, primary microglia) [108,115]
TLR9 [39]	Unmethylated CpG DNA [39]	+ IF, WB, RT-PCR (in vitro, primary neurons) [130]	+ (low) RT-PCR (in vitro, primary astrocytes) Very low on basal level [120], present upon inflammatory stimulation [112,120]	+ WB (ex vivo) [131]	+ RT-PCR (in vitro, primary cells, cell line) [114] − RT-PCR (in vitro, cell line) [113]	+ (low) RT-PCR (in vitro, primary astrocytes) [115]	+ (low) RT-PCR (in vitro, primary microglia) [115]
TLR10 [132]	Triacyl lipopeptides, flagellin [133], EPs, RNA [134]	-	-	-	+ RT-PCR (in vitro, primary neurons) [114] − RT-PCR (in vitro, cell line) [113]	+ (low) RT-PCR (in vitro, primary astrocytes) [115]	− RT-PCR (in vitro, primary microglia) [115] + RNAseq (ex vivo) [135]
TLR11 [43]	Profilin [136]	+ In situ IF (in vivo) [137]	− In situ IF (in vivo) [137]	− In situ IF (in vivo) [137]	-	-	-
TLR12 [138]	Profilin [138]	+ In situ IF (in vivo) [137]	− In situ IF (in vivo) [137]	− In situ IF (in vivo) [137]	-	-	-
TLR13 [44]	Unmethylated bacterial RNA [44]	+ In situ IF (in vivo) [137]	+ In situ IF (in vivo) [137]	+ (low) In situ IF (in vivo) [137]	-	-	-
**Adaptor**	**Connected TLR**	**Expression on CNS-Resident Cells**					
		**Mouse**			**Human**		
		**Neurons**	**Astrocytes**	**Microglia**	**Neurons**	**Astrocytes**	**Microglia**
MyD88 [139]	TLR1, TLR2, TLR4-9, TLR11-13	+ IF (in vitro, primary neurons) [118]	+ WB (in vitro) IF (in vivo, spinal cord astrocytes) [140]	+ RT-PCR (in vitro, cell line), WB (in vitro, primary microglia and cell line) [122,141,142]	+ RT-PCR, WB (in vitro, cell line) [122,143]	+ RT-PCR (in vivo) [144]	+ RNAseq (ex vivo) [135]
TRIF [145]	TLR3, TLR4	+ IF, WB (in vivo, in vitro, primary hippocampal neurons) [146]	+ (low) IF (in vivo) [146]	+ (low) IF(in vivo), WB (in vitro, cell line) [142,146]	+ IR (in vivo) [146]	+ IR (in vivo) [146]	+ IR (in vivo) [146]

## Data Availability

Not applicable.

## Abbreviations

AD	Autosomal dominant
AIM	2-Absent in melanoma 2
ALR	AIM-2-like receptor
AP1	Activator protein 1
AR	Autosomal recessive
BBB	Blood-brain barrier
CBP	CREB binding protein
CCL	Chemokine C-C motif ligand
cGAS	Cyclic guanosine monophosphate-adenosine monophosphate synthase
CLR	C-type lectin receptor
CNS	Central nervous system
COVID-19	Coronavirus disease 2019
CpG	5′-Cytosine-phosphate-Guanine-3′
CREB	Cyclic AMP-responsive element-binding protein
CSF	Cerebrospinal fluid
CXCL	Chemokine C-X-C motif ligand
DENV	Dengue virus
dsRNA	Double stranded RNA
EP	Envelope protein
ER	Endoplasmatic reticulum
FC	Flow cytometry
GP	Glycoprotein
HCMV	Human cytomegalovirus
HeV	Hendra virus
HIV-1	Human immunodeficiency virus 1
HSE	Herpes simplex encephalitis
HSV-1	Herpes simplex virus 1
IAV	Influenza A virus
ICC	Immunocytochemistry
IF	Immunofluorescence
IFN	Interferon
IFNAR	IFN-I receptor
IFN-I	Type-I interferons
IL	Interleukin
iNOS	Inducible nitric oxide synthase
iPSC	Induced pluripotent stem cell
IR	Immunoreactivity
IRAK	IL-1R-associated kinase
IRF	Interferon-regulatory factor
ISG	IFN stimulated gene
ISRE	IFN-stimulated response elements
JAK1	Janus kinase 1
JEV	Japanese encephalitis virus
LGTV	Langat virus
LPS	Lipopolysaccharide
LTA	Lipoteichoic acid
mAb	Monoclonal antibody
MAPK	Mitogen-activated protein kinase
MEF	Mouse embryonic fibroblast
mTOR	Mammalian target of rapamycin
mTORC	mTOR complex
MyD88	Myeloid differentiation primary response 88
NF	κB-Nuclear factor-κB
NiV	Nipah virus
NLR	NOD-like receptor
NOD	Nucleotide-binding oligomerization domain
NS	Nonstructural protein
ODN	Oligodeoxynucleotide
PAMP	Pathogen associated molecular pattern
PBMC	Peripheral blood mononuclear cells
PGN	Peptidoglycan
PKR	Protein kinase R
PRR	Pattern recognition receptor
RABV	Rabies virus
RIG	I-Retinoic acid inducible gene I
RIP1	Receptor-interacting protein 1
RLR	RIG-I like receptor
RNAi	RNA interference
RT	PCR-Reverse transcription polymerase chain reaction
SARS-CoV-2	Severe acute respiratory syndrome coronavirus 2
sfRNA	Subgenomic flavivirus RNA
ssRNA	Single stranded RNA
STAT	Signal transducers and activators of transcription
STING	Stimulator of interferon genes
TBE	Tick-borne encephalitis
TBEV	Tick-borne encephalitis virus
TBK1	TANK-binding kinase 1
TCA	Tricarboxylic acid
TLR	Toll-like receptor
TMEV	Theiler’s murine encephalomyelitis virus
TNF-α	Tumor necrosis factor α
TRAF	TNF receptor-associated factor
TRIF	TIR-domain-containing adapter-inducing interferon-?
TYK2	Tyrosine kinase 2
VSV	Vesicular stomatitis virus
VZV	Varicella-zoster virus
WB	Western blot
WNV	West Nile virus
WNV-E	WNV envelope protein
ZIKV	Zika Virus
